# Unveiling the Graphite Electrolyte Interphase Evolution under Fast Charging Conditions in Commercial Cells

**DOI:** 10.1021/acsami.5c17267

**Published:** 2025-12-02

**Authors:** Alex Liu, Weikang Li, Bing Han, Phillip Ridley, Louis Ah, Bhargav Bhamwala, Marta Vicencio, Dhevathi R. R. Kannan, Vallabha R. Rikka, Vinay Premnath, Judith A. Jeevarajan, Wurigumula Bao, Ying Shirley-Meng

**Affiliations:** † Aiiso Yufeng Li Family Department of Chemical and Nano Engineering, 8784 University of California, San Diego, La Jolla, California 92093, United States; ‡ Pritzker School of Molecular Engineering, University of Chicago, Chicago, Illinois 60637, United States; § Underwriters Laboratories Research Institutes, Electrochemical Safety Research Institute, Houston, Texas 77204, United States

**Keywords:** lithium-ion battery, fast-charging, electrodes, solid electrolyte interphase, lithium iron phosphate, graphite, characterization

## Abstract

The resurgence of LiFePO_4_ lithium-ion batteries as a competitive alternative to nickel–cobalt systems for electric vehicle (EV) applications, driven by their superior thermal stability and cycle life, necessitates a thorough understanding of their degradation modes to develop strategies for performance and safety enhancements. This study investigates cycling-induced degradation in 18650 LiFePO_4_/graphite full cells at varying charge rates. We analyze capacity degradation mechanisms through electrochemical performance, surface and bulk morphology, composition, and structure of both the cathode and anode. Our results reveal that irreversible lithium loss, primarily due to solid-electrolyte interphase formation, dominates at lower charging rates. However, above 4C, graphite electrode degradation is distinct and limited by Li-ion intercalation kinetics. Notably, degradation mechanisms vary not only with charging rate but also spatially across the graphite electrode. This work highlights the degradation mechanisms of commercial LiFePO_4_/graphite systems under high charge rates, providing insights into critical bottlenecks in lithium-ion battery development for fast-charging applications.

## Graphical abstract

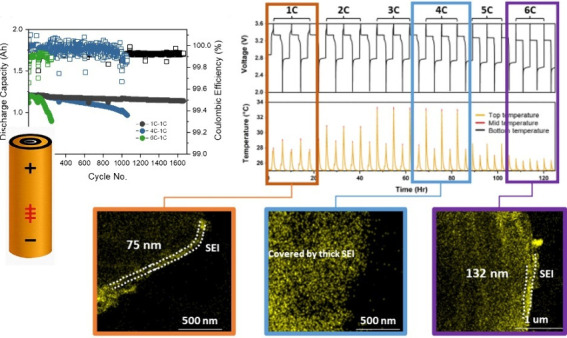

## Introduction

Lithium-ion batteries (LIBs) have been at the forefront of applications pertaining to consumer electronics in the past few decades, and the global demand for high-energy secondary batteries has only continued to grow. This is especially true in the transportation sector, where electric vehicles (EVs) have been proposed as a particular solution to the need for rapid electrification^1,2^ which can lead to improved decarbonization and a healthier environment. Among a host of cathode materials, olivine LiFePO_4_ (LFP) has stood out for its high thermal stability and low cost when compared to cobalt-containing chemistries, as well as exceptional cycling stability, and benignity to the environment. On the other hand, the main drawbacks found with LFP are its relatively low energy density, poor lithium diffusion and poor electronic conductivity.^3^ However, nanostructuring of LFP as well as carbon coating have been utilized to mitigate the diffusion and electronic conductivity limitations,^4–6^ whereas fast charging of LFP/Graphite (Gr) LIBs may be an effective method of overcoming range anxiety due to LFP’s relatively low energy density. As such, research on the degradation mechanisms of performance loss under high charge current rates in LFP/Gr batteries becomes essential.

Prior research conducted on the causes of capacity loss in LIBs during long-term cycling spanning thousands of cycles have suggested similar cycling-induced degradation mechanisms: (1) lithium inventory loss; active material loss at the (2) negative electrode or (3) positive electrode.^7–9^ In this system, the LFP cathode is the only source of active lithium, and capacity losses associated with the cathode necessarily stems from structural change, excessive cathode electrolyte interphase (CEI) formation, or active material loss. As LFP operates at a relatively low potential compared with other LIB cathodes, electrolyte oxidation is not as severe as in layered oxide cathode-based LIBs. Nevertheless, CEI evolution contributes to LFP LIB degradation as a heterogeneously thick CEI may cause a loss of electrical contact within the electrode and at the electrode-current collector interface. Regarding active material loss, Amine et al. demonstrated that iron dissolution from LFP strongly correlated with capacity loss, which was mainly associated with an ion exchange between LFP and protons from generated HF in conventional LiPF_6_-based carbonate electrolytes.^10^ Coordinated Fe^2+^ was proposed by Dahn et al.^11^ to migrate and reduce on the Gr anode surface as Fe^0^, potentially inhibiting Li^+^ intercalation.

Meanwhile, capacity losses associated with Gr anodes are primarily dominated by excessive solid-electrolyte interphase (SEI) formation, unwanted Li-metal (Li^0^) plating, and inactive lithiated Gr trapping (Li_*x*_C_6_).^12–15^ Unwanted Li^0^ plating on Gr anodes poses a challenge to reversible fast charging of LIBs and is generally believed to stem from the slow kinetics of the Li-ion intercalation process into Gr.^16,17^ Wang et al. demonstrated that, as opposed to Li^0^ plating on Gr anode necessarily due to kinetic limitations of the Gr anode, thermodynamics-induced Li^0^ plating can also occur with the formation of a sufficiently large temperature gradient due to the temperature dependence of the equilibrium electrode potential.^18^ While some previous studies have focused on the accelerated degradation mechanisms of LFP/Gr LIBs under fast rates of discharge,^19–21^ a comprehensive understanding on the degradation mechanisms under fast rates of charge remains limited. This is largely due to the difficulty in decoupling lithium inventory loss from deposited Li^0^ and inactive Li_*x*_C_6_. The formation of these two sources of lithium inventory loss may occur in parallel with the formation of Li_*x*_C_6_ exaggerated by the volume expansion of plated Li^0^ during the charging process. Clearly, there is a complex interplay between the LFP cathode and Gr anode which makes a holistic degradation mechanism challenging, especially considering the cycling-condition dependence of the available degradation mechanisms to both electrodes.

Various characterizations have been employed to elucidate the capacity decay in LIBs quantitatively spanning diffraction methods to mass spectroscopy. Liu et al. employed *in situ* X-ray diffraction (XRD) on 18650 LFP LIBs to monitor the microstructural changes in the LFP cathode at a medium charge/discharge rate of 1 C, revealing that the loss of active lithium in the system is the primary cause of capacity fading as the diffraction pattern contour over 2500 cycles remains nearly symmetric.^20^ McShane et al. utilized mass spectrometry titration techniques to quantify inactive Li^0^/Li_*x*_C_6_, and SEI on the Gr anode under fast rates of charging (4C), demonstrating that plated Li^0^ induces excessive SEI growth.^22^

Herein, we focus on the cycling-induced degradation mechanisms of 18650 LFP/Gr commercial cells under high rates of charge (4C, 6C), corresponding to fast charging – defined by the United State Advanced Battery Consortium as reaching 80% state of charge within 15 min under constant current —and equivalent discharge rates (1C). Titration gas chromatography (TGC) was employed to quantify and segregate the plated Li^0^ from the inactive Li_*x*_C_6_ at the Gr anode as protic solvents will react with the two to evolve H_2_ gas whereas SEI will not. Electron microscopy was utilized to investigate the local microstructural evolutions on both electrodes with application of X-ray photoelectron spectroscopy (XPS) and electrochemical impedance spectroscopy (EIS) to probe the growth in electrolyte-electrode interphase. By coupling quantification of the lithium inventory to the chemical composition of the electrode surfaces, along with observations of local anode microstructures in electron microscopy, we elucidate the SEI evolution with rates of charge and electrode locality. The results show that the continuous SEI formation over time is the primary cause of capacity decay and is further locally influenced by the temperature gradient across the cell at higher rates of charge.

## Experimental Section

### Materials

18650 LiFePO_4_/graphite batteries were purchased from A123/LithiumWerks Systems. The selected batteries were rated at a nominal capacity of 1.2 Ah. Prior to cycling tests, all received batteries were discharged at a rate of 1C due to being shipped at a nominal voltage of 3.3 V. Subsequently, the exact capacities of the cells were measured at a charge/discharge rate of 1C under room temperature to verify the consistency of the specified nominal capacity. The applied currents for fast-rate charging are thus based on the nominal capacities measured, with the batteries operating between a voltage range of 2.0 and 3.6 V.

### Electrochemical Testing

Commercial Gr||LFP 18650 cells were first predischarged at 1C (1.2 A) from open circuit voltage to 2.0 V prior to electrochemical cycling. Following predischarge, the cells were cycled between 2.0 and 3.6 V at different rates of charge (1C, 4C, and 6C) and the same rates of discharge (1C) based on the nominal capacity of 1.2 Ah. The same rate of discharge was applied on all cells to isolate and identify the effect of charging rate on degradation. A constant current – constant voltage cycling protocol was utilized with galvanostatic cycling from 2.0 V up to 3.6 V, and potentiostatically held at 3.6 V until the current decayed to C/20 (0.06 A). The electrochemical cycling of all cells was conducted at room temperature by an Arbin BT2000 cycler (Arbin instrument, USA), with testing terminated upon reaching 80% of the nominal capacity (0.96 Ah).

Symmetrical LFP cathodes and Gr anodes are fabricated from the disassembled 18650 cells with 0.5-in. diameter punched electrodes, Celgard 2325, and LP57 electrolyte. EIS is conducted on an SP-200 Biologic potentiostat with a 10 mV sinusoidal AC voltage perturbation from an initial frequency of 1 MHz to 100 mHz. The resulting impedance spectra are subsequently fitted by equivalent circuit models on ZView.

### Characterizations

#### Titration Gas Chromatography

TGC experiments were conducted with a Nexis GC-2030 Gas Chromatograph (Shimadzu). Two samples of 0.5-in. diameter are each punched from the center and edges of the graphite and separator as disassembled from the electrochemically cycled 18650 LFP/Gr cells. The glovebox pressure was then lowered to 1 atm and the samples were sealed inside of 30 mL flasks with rubber septa and electrical tape. One mL of deionized water was injected to react the inactive metallic Li to form Hydrogen gas. Flasks with duplicate samples were similarly injected with 1 mL of deionized water but followed by 1 mL of 3 M H_2_SO_4_ solution as well. All flasks were well mixed by shaking, and 30 μL of resulting gas was injected into the chromatograph via a gastight Hamilton syringe for the H_2_ gas measurement. Utilizing a calibration curve (Figure S5), the mass of inactive metallic Li and inactive Li_*x*_C_6_ can be quantified by the H_2_ peak areas. Subsequently, the mass values are utilized to calculate the areal capacities of both inactive metallic Li and inactive Li_*x*_C_6_ present in the middle and edge areas of the graphite anodes in the cycled cells.

#### X-ray Photoelectron Spectroscopy

XPS was conducted on Kratos AXIS-Supra, utilizing an Al X-ray source under 10^–9^ Torr. The cycled LFP cathodes and Gr anodes were first rinsed with DMC to remove residual salts and electrolyte. Survey scans were performed with a step size of 1.0 eV, followed by a fine scan with 0.1 eV resolution. All spectra were analyzed by CasaXPS software for chemical species identification.

#### Nuclear Magnetic Resonance (NMR)

NMR was conducted on electrolytes to analyze the salt species in the electrolyte. The NMR measurements of the electrolyte samples were performed with a Jeol ECA 500 spectrometer. Electrolytes from cylindrical cells were collected and mixed with anhydrous deuterated dimethyl sulfoxide (d-DMSO) to form a clear solution. The NMR sample was then sealed in an NMR tube under Ar prior to measurement. Resultant NMR spectra were analyzed with MestReNova.

#### Cryogenic FIB-SEM

After cycling, the 18650 cells were disassembled in an Ar-filled glovebox to obtain the Gr anode and LFP cathode. The samples were then washed with DMC to remove residual electrolyte and salt. The sample was mounted on an SEM stub in the glovebox and then transferred to a FEI Scios DualBeam FIB-SEM system using an airtight transfer holder to limit air exposure. The sample stage was cooled to −180 °C using liquid nitrogen to minimize beam damage to deposited lithium on the Gr anode. The Gr sample cross-section was milled with a gallium ion beam using a voltage of 30 kV, a current of 7 nA, and a dwell time of 100 ns. The cross-section was cleaned with the gallium ion beam at a reduced current of 1 nA. SEM images of the cross-section were taken using an Everhart–Thornley detector at 5 kV and 0.1 nA.

#### Transmission Electron Microscopy

TEM was performed utilizing Thermofisher Talos 200X at 200 kV. The cycled LFP cathodes and Gr anodes were firstl rinsed with DMC to remove residual salts and electrolyte, and electrodes were scraped onto a TEM Cu grid. STEM-based energy-dispersive X-ray spectroscopy (STEM-EDS) was performed with 4 in-column SDD Super-X detectors also operating at 200 kV, with a probe current of approximately 140 pA and an acquisition time of 3 min for EDS mapping.

#### X-ray Diffraction

XRD diffraction patterns were collected on a Bruker APEX II Ultra diffractometer with a Mo X-ray source (λ = 0.71073 Å). The LFP cathode and Gr anodes were first rinsed with DMC to remove residual salts and electrolyte, and prepared by scratching the electrodes to fill thin-walled capillary tubes inside an Ar-filled glovebox (<0.1 ppm of H_2_O, < 0.1 ppm of O_2_). Rietveld refinement is conducted on the General Structure Analysis System II (GSAS-II) utilizing crystallographic data from ICSD.

#### Inductively Coupled Plasma–Mass Spectrometry (ICP-MS)

ICP-MS analysis was performed with a Thermo iCAP RQ ICP-MS to analyze the elemental concentration of Fe on electrode surfaces. Electrodes were digested in a mixture of H_2_SO_4_ and H_2_O_2_ at a volume ratio of 1:1 over a few days prior to dilution.

#### Fiber Bragg Grating Sensors

FBG optical sensors (5 mm) from Samyon were calibrated in a temperature range of 15–55 °C using a Memmert IPP55 climate chamber with 10 °C steps and 4h intervals. The sensors were attached to an 18650 Gr-LFP cell for surface thermal measurements within a thermally isolated box under a static 25 °C in the same climate chamber.

## Results and Discussion

### Cycling Tests

A comparative study on the electrochemical performance of the 18650 LFP/Gr Li-ion batteries was conducted to study the cycling induced degradation mechanisms under high rates of charging. Owing to the commercial nature of the cells, all cells in the study underwent prescreening to verify a consistent nominal capacity of 1.2 Ah. All cycling was performed with constant current-constant voltage (CC–CV) charge, with a voltage range of 2.0 to 3.6 V. CV is employed at the end of the charging process at 3.6 V until the current drops to 0.05 C. Besides a typical current cutoff on the CV command of the testing protocol, a time limit is imposed as practical application of the batteries under fast-charging conditions would necessitate a reasonable total charge time, accounting for CV. Hence, time limits of 3600, 900, and 600 s were included as an alternative termination limit to a taper current threshold of C/20 for charging C-rates of 1C, 4C, and 6C, respectively. The details of cycling are summarized in the following Table 1.

**Table 1:** Testing Protocol of 18650 LFP/Gr Cells^a^

#	Command	Parameter	Limits	Registration
**1**	Rest	–	*t* ≥ 1800 s	*t* = 2 s
**2**	Charge	CC @ 1C/4C/6C	V > 3.6 V	*t* = 1 s OR dV = 0.01 V
**3**	Charge	CV @ 3.6 V	I ≤ C/20 OR *t* = 3600 s/900 s/600 s	*t* = 1 s OR dI = 0.001 A
**4**	Discharge	CC @ 1C	V ≤ 2.0 V	*t* = 1 s OR dV = 0.01 V
**5**	Loop	To #2	80% of nominal capacity (1.2 Ah)	

a C-rates are based on the nominal capacity of 1.2 Ah as specified by the cell manufacturer.

Maintaining the voltage at 3.6 V becomes crucial to eliminating potential sources of polarization. As shown in Figure 1a, each cell showed a characteristic plateau at the average voltage of 3.4 and 3.2 V for the charge and discharge steps, respectively. A consistent nominal capacity of 1.20 Ah was obtained for all 8 cells with an average initial Coulombic efficiency (CE) of 99.67%. Subsequently, long-term cycling under CC–CV at charging rates of 1C, 4C, and 6C with a fixed discharge rate of 1C were carried out until 80% of the 1.2 Ah nominal capacity was reached. The discharge rate was kept constant at 1C to investigate the cycling-induced degradation of the system under fast-charging conditions, specifically. Figure 1b. illustrates the trend of the discharge capacity and CE with cycles for the cells charged at 1C, 4C, and 6C. In stark contrast to charging at a relatively low rate of 1C, cells charged at 4C and 6C not only reached the 80% capacity retention cutoff more quickly (4C = 1059 cycles; 6C = 246 cycles) but also displayed more unstable CE. Upon closer inspection, inflection points in which capacity begins to drop for the cells charged at 4C and 6C are observed at 200 and 150 cycles, respectively. Moreover, a second inflection point was observed for both cells at 1000 and 200 cycles for the cells charged at 4C and 6C, respectively. Compared to the cell charged at 1C, It is clear that the on these inflection points occur earlier with increased charging C-rates, suggesting different degrees of degradation kinetics. Presence of inflection points suggests a transition in dominant degradation mode or an onset of accelerated degradation. While the cell charged at 4C displayed a high average CE (99.97%), it is important to note that this is a consequence of an unstable CE with CE’s over 100%. The unstable CE for both the cells charged at 4C and 6C indicates the instability of the redox reactions and irreversible reactions of the active lithium, either on the cathode or anode side.^23^ Conversely, the cell charged at 1C shows a very low rate of degradation with an average CE of 99.93% and a capacity retention of 95.08% even at 1663 cycles.

**Figure 1:**
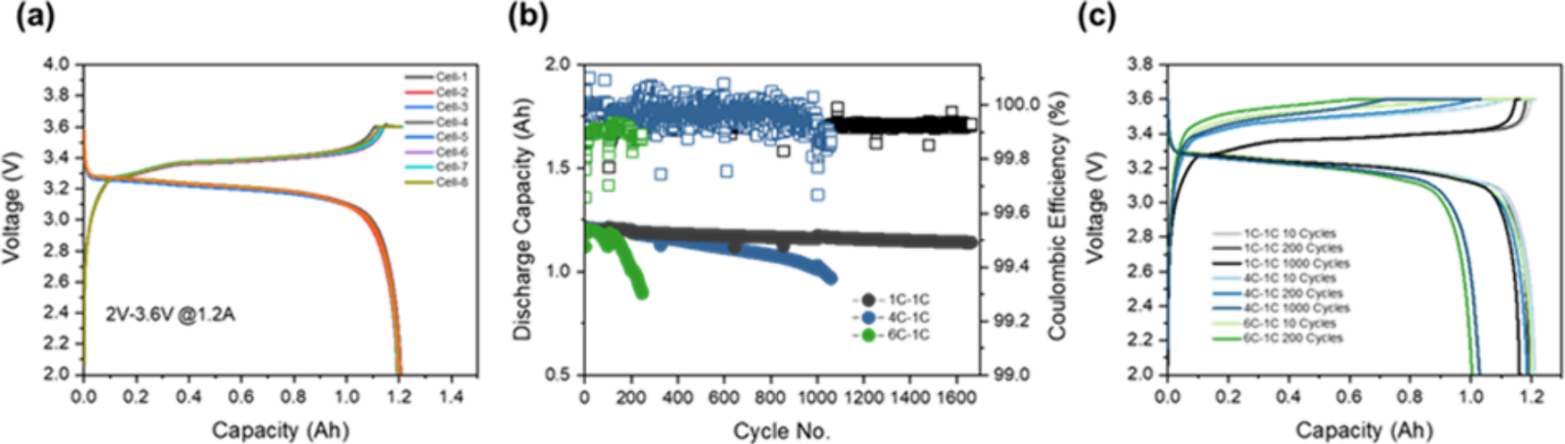
(a) 1st cycle performance of the LFP/Gr cell at 1C-1C. (b) Cycling performance and (c) charge and discharge curves of LFP/Gr cell at 1C-1C, 4C-1C, and 6C-1C.

Figure 1c shows the voltage–capacity curves of the fast-charging cycles at 10, 200, and 1000 cycles. When examining the evolution of voltage hysteresis between the charge and discharge plateaus over long-term cycling, we observe that the increase in discharge voltage hysteresis for cells charged at 1C and 4C is relatively minor over 200 cycles (4.42 mV), compared to a significantly larger increase for cells charged at 6C (33.70 mV). This pronounced hysteresis at higher charge rates indicates notable polarization effects or elevated internal impedance within the cell. Additionally, a substantial portion of the charge capacity for the 6C-charged cells arises from the constant voltage (CV) step, a trend that becomes apparent from the onset of cycling. For example, in the first cycle, the CV step accounts for 34% of the total charge capacity. As the cells have undergone many cycles, this behavior is not ascribed to an excessively thick SEI. Rather, it is ascribed to limited Li^+^ charge transfer kinetics at the Gr/electrolyte interphase, which has been reported to be much lower than that of the LFP/electrolyte interphase.^24^ After 200 cycles, this contribution increases only marginally, reaching 39% of the total charge capacity.

In contrast, the increase in CV contribution to charge capacity at 4C is more gradual and delayed, rising from 13% at 200 cycles to 32% at 1000 cycles, along with a corresponding increase in voltage hysteresis from 4.42 mV to 32.41 mV. This delayed onset and eventual higher contribution suggest a degradation mechanism that is distinct from that observed at 6C. The gradual increase may be attributed to physical degradation of the electrodes, likely caused by higher concentration gradients within the electrode particles at elevated C-rates, which in turn induce greater diffusion-induced stresses.^24–26^ While kinetic limitations of the graphite anode are likely also involved, as is the case for 6C charging—the later onset and larger magnitude of CV contribution at 4C imply a fundamentally different degradation pathway.

### Morphological Analysis and Li-Inventory Quantification

After long-term cycling to 80% capacity retention based on the 1.2 Ah nominal capacity, the 4C and 6C charged cells were disassembled for post-mortem analysis to better identify the mechanisms of capacity loss. As baseline reference, a cell cycled at a symmetric rate of 1C for 1 cycle was also taken apart. Notably, visual analysis of the Gr anode showed distinct discoloration between the center and edges of the electrode sheet at 4C and 6C. However, the degree of discoloration difference between the center and edges of the electrode sheet is much more apparent in the 4C cell, along with gray deposits of Li-metal (Figure S1a-c). Although discoloration of the edges of negative electrodes is typically expected as they are generally larger than the positive electrodes, the boundaries observed here are much larger. Conversely, the LFP cathode did not display any specific damage after the cycle life testing. Optical analysis (Figure S 1d-f) did not reveal any specific deposits or mechanical stresses, such as cracking or exfoliation on the electrode surface. The 4C and 6C LFP cathodes retain a glossy surface much like the baseline 1C LFP cathode. Figure S2a-c shows the SEM morphologies of the LFP cathodes, depicting no obvious difference in the morphologies at both 4C and 6C relative to the baseline. As carbon coating of LFP is commonly used to enhance the electronic conductivity of LFP beyond the typical carbon additives, attenuation of the carbon coating layer could impact cell performance, particularly at high C-rates. However, TEM results show that the nanometer-thick carbon coating on cycled LFP particles remain intact (Figure S3).

Owing to the obvious differences in the macroscopic features observed on the Gr anode at 4C and 6C, along with the differences between the center and edges of the electrode sheets, Cryo FIB-SEM was employed to probe both the surface and bulk morphologies. Gr particles are uniformly distributed across the surface of the 1C first cycle Gr anode (Figure 2a,c) with a compact cross-section (Figure 2b,d). While no significant morphological differences are seen between center and edge at either the surface or bulk of the baseline 1C first cycle sample, considerably different morphologies are observed on the cycled Gr anodes at higher rates of charge. A thick and electronically insulating interlayer had appeared on the surface of the 4C Gr anode at the center, highlighted by the contrast of the surface and cross-sectional SEM images, depicted in Figure 2e,f. Meanwhile, the Gr particles on the edge surface were observed to be flattened while the cross-section appears relatively more porous (Figure 2g,h). On the contrary, the surface and bulk of the 6C Gr anode at the center appears identical to the 1C first cycle reference, with uniformly dispersed granular Gr particles (Figure 2i,j) whereas the edge surface appears densified with an electronically isolating layer penetrating a few μm from the surface into the bulk (Figure 2k,l).

**Figure 2:**
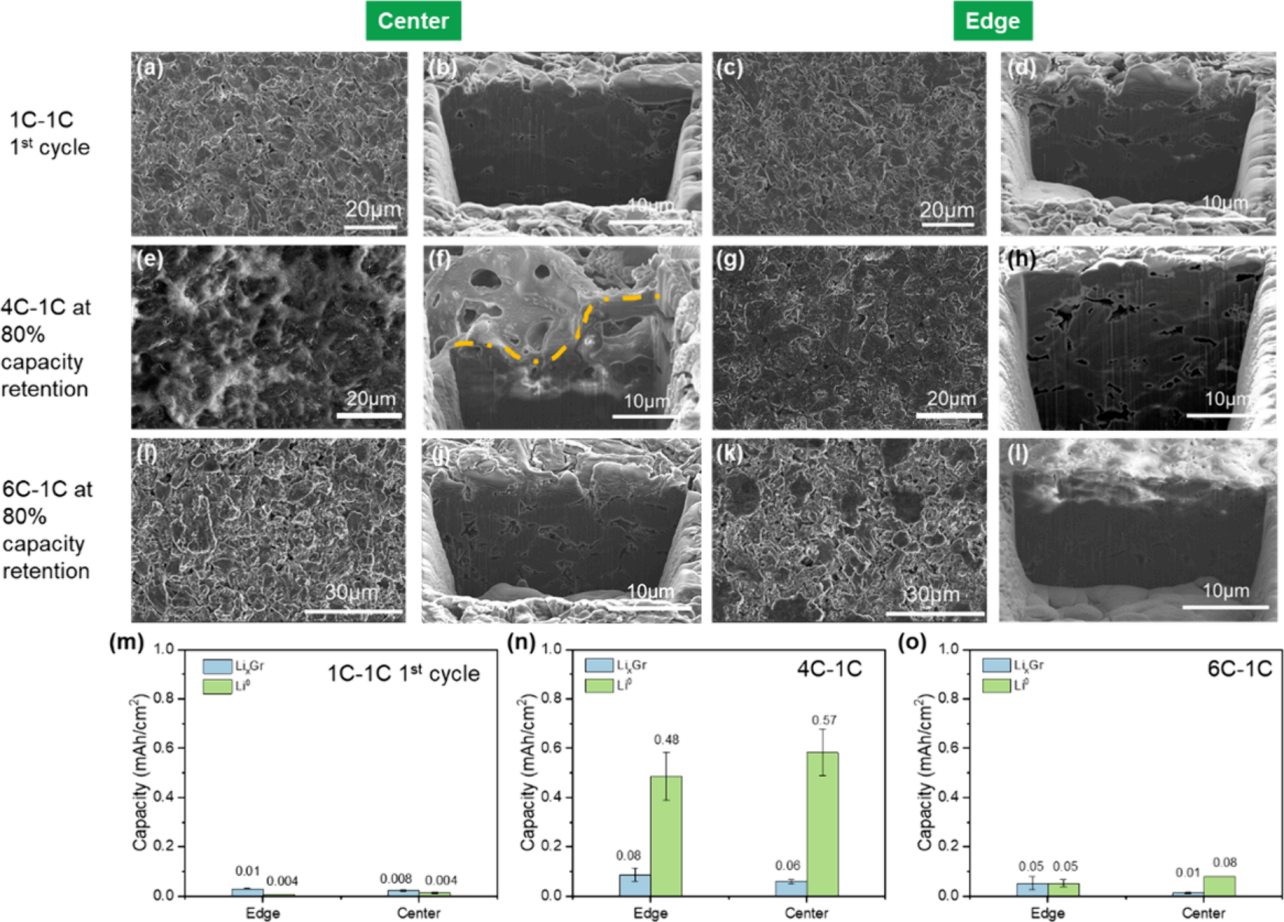
(a–d) Surface and cross-section SEM image of cycled Gr at 1C-1C 1st cycle. (e–h) Surface and cross-section SEM image of cycled Gr at 4C-1C after 80% capacity retention. (i–l) Surface and cross-section SEM image of Gr cycled at 6C-1C after 80% capacity retention. (m–o) Li^0^ and Li_*x*_C_6_ quantification of cycled Gr at 1C-1C 1st cycle, 4C-1C, and 6C-1C.

The densification of the Gr electrode is attributed to the continuous SEI growth, plating of Li^0^ over time, or both. Surface examinations reveal a higher degree of densification in the surface edge of the 4C Gr anode compared to the surface edge of the 6C Gr anode, indicating a different dominant mechanism for the compaction between the two charging rates. Locally, the heterogeneity of the Gr densification indicates the heterogeneity of Li^0^ plating, aligning with previous reports on the heterogeneous local current densities at charging rates as high as 6C.^27,28^ Likewise, cross-sectional examination of the anode which reveals a difference in the degree of porosity change between 4C and 6C may be explained by the difference in cycle life for the given failure criteria of 80% capacity retention between 4C and 6C (1200 cycles vs 200 cycles). Regardless, the interfacial evolution of the Gr anode in the LFP/Gr system evidently differs locally between 4C and 6C based on the SEM images. To further isolate the capacity loss mechanism, XRD was applied to both the Gr anodes and LFP cathodes (Figure S4). Comparing the characteristic diffraction patterns of the 4C and 6C electrodes to 1C first cycle, no obvious changes are seen, demonstrating that the fast-charging rate has not been critically detrimental to the material structure itself.

As such, quantifying the amount of inactive Li within the cycled Gr anode becomes crucial to understanding the capacity loss associated with fast charging conditions. Inactive Li on the cycled Gr anode typically exists as Li-containing SEI compounds (SEI Li^+^). In the case of fast charging, plated Li^0^ may also exist on the Gr anode surface, which may be passivated by electrolyte, forming more SEI and potentially becoming electronically isolated from the Gr anode. Li^0^ plating on Gr often coincides with the formation of inactive Li in the form of Li_*x*_C_6_, likely a direct consequence of the high volumetric expansion associated with Li^0^ plating under fast charging conditions.^29^ Utilizing titration gas chromatography (TGC), Li^0^ as well as inactive Li_*x*_C_6_ may be quantified.^30^ While both sources of Li can react with protic solvents to form hydrogen gas (H_2_), segregation of the two is achievable via the utilization of two different protic solvents with differing Lewis acidity with reaction pathways succinctly listed in Table S1. Unlike Li^0^, which may be fully titrated by H_2_O, Li_*x*_C_6_ is only partially titrated. Consequently, a 3 M solution of H_2_SO_4_ is injected 15 min after the injection of H_2_O to the cycled Gr anode to fully titrate the remaining Li_*x*_C_6_. The choice of concentration was determined in a previous study,^31^ as >3 M solutions of H_2_SO_4_ induced additional side reactions with the Cu current collector on the Gr anode, impacting the titration accuracy. Furthermore, Bao et al. developed and verified a linear relationship between the capacity associated with Li_*x*_C_6_ and generated H_2_ amount utilizing 3 M H_2_SO_4_ solution.^31^

Based on this calibration curve (Figure S5), accurate quantification of the Li^0^ and inactive Li in the form of Li_*x*_C_6_ in Gr anodes cycled under fast charging rates are achieved. The summarized areal capacities corresponding to the Li^0^ and Li_*x*_C_6_ are summarized in Figure 2m-o for 1C first cycle, 4C, and 6C, respectively. As the 1C Gr sample only underwent a single cycle, a negligible amount of Li^0^ and Li_*x*_C_6_ were detected as expected as shown in Figure 2m. In comparison, an exceedingly large amount of Li^0^ was detected on the 4C Gr anode, which was relatively higher in the center, depicted in Figure 2n. Pore clogging of Gr electrodes due to SEI growth has been described by several studies,^31–35^ negatively impacting the electrode kinetics due to reduced electrode porosity, subsequently increasing electrode tortuosity and likeliness of Li-metal plating. The relatively large amount of Li^0^ detected with the 4C Gr suggests a similar mechanism for the onset of Li-metal plating over long-term cycling. Thickness measurements of the cycled Gr electrodes are summarized in Table S2, where the ∼52% thickness increase of the 4C Gr at the center corroborates with the excessive SEI growth depicted by FIB-SEM. As for the 6C Gr anode, it is noteworthy that a similar amount of Li_*x*_C_6_ was found in the electrode edge compared to the 4C electrode center and edge, but 5-fold the amount compared to the center, shown in Figure 2o. This local discrepancy is investigated and discussed in the following discussion.

### Interfacial Analysis on the Graphite Anode

STEM-EDS and XPS analysis of the Gr anodes further supports the TGC results. Figure 3a and Figure S6 shows the morphology of the Gr anode via elemental mapping of F, O, C, and P. After a single cycle at a charge rate of 1C, a SEI layer of ∼75 nm is observed by TEM. At higher charge rates of 4C and 6C, however, thicker and uneven SEI ranging from 132 nm to >1 μm are observed. The thickness of the SEI was notably higher locally for both the center and edge of the Gr anode charged at 4C compared to 6C, indicating a large amount of active Li consumption on the anode interphase. To verify the primary source of active Li inventory loss by interphase growth on the anode, XPS was carried out on the corresponding LFP cathodes, where the presence of C 1s and O 1s signals is attributed to solvent decomposition with cycling, and F 1s and P 2p indicate salt decomposition (Figure S7). Evidently, no significant changes are observed in CEI chemistry after charging at 4C and 6C when compared to 1C. Similarly, XPS depth profiling was applied to understand the distribution of elements within the SEI matrix with charging rates, as highlighted in Figure 3b and Figure S8–10. Furthermore, the electrolyte primary constituents were probed by nuclear magnetic resonance (NMR) for further insight into sources of decomposition as inferred by XPS. LiPF_6_ was confirmed as the salt by both ^19^F and ^31^P spectra (Figure S11a,b), and the primary solvents were identified as ethylene carbonate (EC), diethyl carbonate (DEC), and ethyl methyl carbonate (EMC) based on the ^13^C and ^1^H spectra (Figure S11c,d). The Li 1s and O 1s spectra are focused on as the differences between the samples at both center and edge are predominantly based on SEI composition, which for typical dilute carbonate electrolytes are composed of inorganic Li_2_CO_3_, Li_2_O, LiF, and organic ROLi species.^36^ Indeed, these characteristic SEI components are supported by the C 1s and F 1s spectra in Figure S8 after a single cycle at 1C. Compared to the cycled Gr anode charged at 6C, the appearance of POF_3_ is detected at 4C. This decomposition product is triggered by LiPF_6_ decomposition and may trigger cascade reactions with carbonate solvents.^37^ Given the high propensity for LiPF_6_ salts to react with trace moisture, the resultant byproducts are generally LiF, POF_3_ and HF. Complementary to the detection of POF_3_ on the 4C Gr anode is the Li 1s and F 1s spectra (Figure S9), which depicts persistent LiF content with etching time, further verifying the high degree of electrolyte decomposition and SEI formation over cycling. It is likely that along with higher LiF and POF_3_ decomposition products observed on the 4C Gr anode, that a higher amount of corrosive HF was generated.^38^ This is in good alignment with the relatively high degree of elemental Fe (Figure S12) observed on the 4C anode surface via ICP-MS, attributed to HF corrosion of the LFP cathode. While no notable differences are observed between the XPS spectra for the 6C Gr anode (Figure S10) compared to single cycled Gr anode at 1C, it is noteworthy that the degree of SEI formation varied locally between the center and edges of the electrode (132 nm at center vs 234 nm at edge). This is contrasted by the opposite trend observed at 4C, where a thinner SEI can be seen on the edge (>1 um at center vs 300 nm at edge). Differences in SEI thickness at the center of the graphite (Gr) anode between the two charging rates are consistent with the observed contributions of the CV step to the total charge capacity. As previously noted, the CV contribution increased from 34% to 39% for the cell charged at 6C, and from 13% to 32% for the cell charged at 4C, as the utilized capacity approached 80% of the nominal 1.2 Ah capacity. The significant increase in CV contribution for the 4C-charged cell is attributed to continuous SEI formation during long-term cycling, which impedes charge-transfer kinetics.

**Figure 3:**
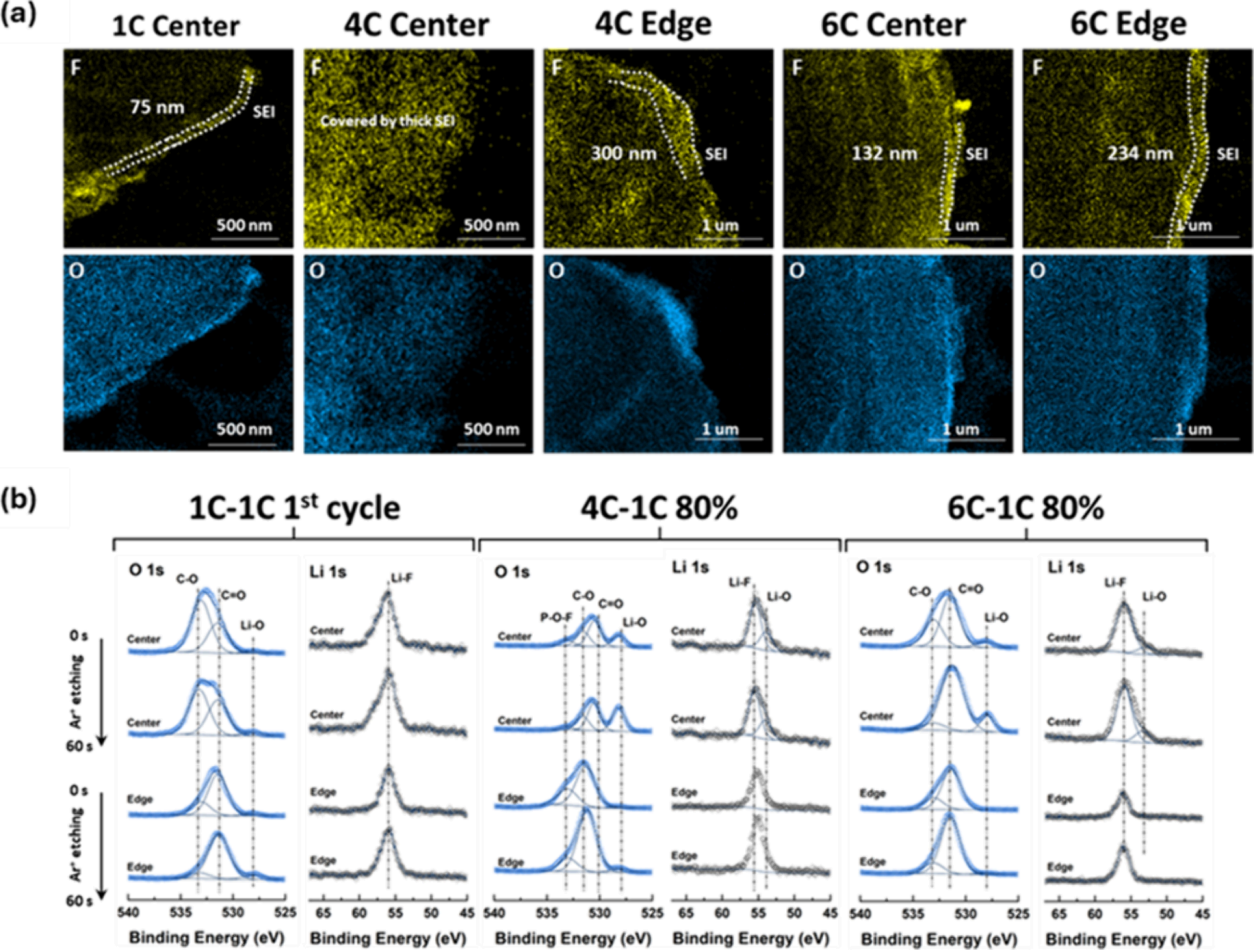
(a) STEM-EDS results of F and O along with (b) O 1s and Li 1s XPS spectra of cycled Gr at 1C-1C 1st cycle, 4C-1C, and 6C-1C.

In contrast, the thinner SEI layer observed at the center of the anode in the 6C-charged cell in conjunction with the smaller change in CV contribution suggests that, although high C-rates also promote ongoing SEI growth, the primary limitation to capacity retention in this case arises from kinetic barriers to Li^+^ intercalation into the graphite anode. Furthermore, the lower total number of cycles completed by the 6C-charged cell (253 cycles) compared to the 4C-charged cell (1060 cycles) likely resulted in less overall SEI accumulation. Moreover, discrepancy in SEI homogeneity is linked not only to rates of charging, but also to pressure and heat generated as it is well-known that these physical phenomena vary across the 18650-cell length and can be largely different between the outermost and innermost portions of the cell roll.^39–41^

To distinguish the cathode impedance growth from the full cell, symmetrical Gr||Gr coin cells were fabricated with conventional 1 M LiPF_6_ in EC/EMC (3:7 wt %) from the cycled 18650 Gr-LFP cells and subjected to electrochemical impedance spectroscopy (EIS). An equivalent circuit comprising of a resistor R_1_ corresponding to the ohmic resistance of the cell components and electrolyte (R_s_), R_2_ corresponding to the charge transfer resistance (R_ct_) through the Li^+^ transfer through the electrode–electrolyte interface, CPE_1_ corresponding to the double layer capacitance, and the Warburg diffusional resistance element W_s1_ is utilized to fit the experimental EIS data. To substantiate the differences in local SEI morphology for the Gr anodes charged at 4C and 6C, separate sets of Gr||Gr symmetrical cells were fabricated from the corresponding locations of the 18650 Gr-LFP anodes. For both the center and edges of the Gr anodes, the impedance spectra corresponding to single cycled Gr anode at 1C and 6C (80% capacity retention) were only fitted with a single RC combination, whereas Gr cycled at 4C to 80% capacity retention was fitted with 2 RC combinations owing to the presence of two partially overlapping semicircles (Figure 4a,b). This may be explained by the Gr not being fully delithiated, as Xu et al. previously reported that EIS of typical Li||Gr cells at voltages above 0.8 V (fully delithiated) show no secondary semicircle characteristically attributed to the charge-transfer process as no further electrochemical reactions occur.^42^ A significantly higher charge transfer resistance is observed for the Gr anode center cycled under a charge rate of 4C compared to the edge (Figure 4c,d), whereas the opposite is true for the Gr anode cycled at 6C, albeit with a marginal difference. As a cross-reference, the EIS of symmetrical cells comprising LFP cathodes from the 18650 Gr-LFP cell were conducted under identical conditions. The results depict low charge transfer resistance (R_ct_) at 4C and 6C, shown in Figure S13, compared to the corresponding Gr anodes, validating the active Li inventory loss from interphase growth as stemming from the Gr anode.

**Figure 4:**
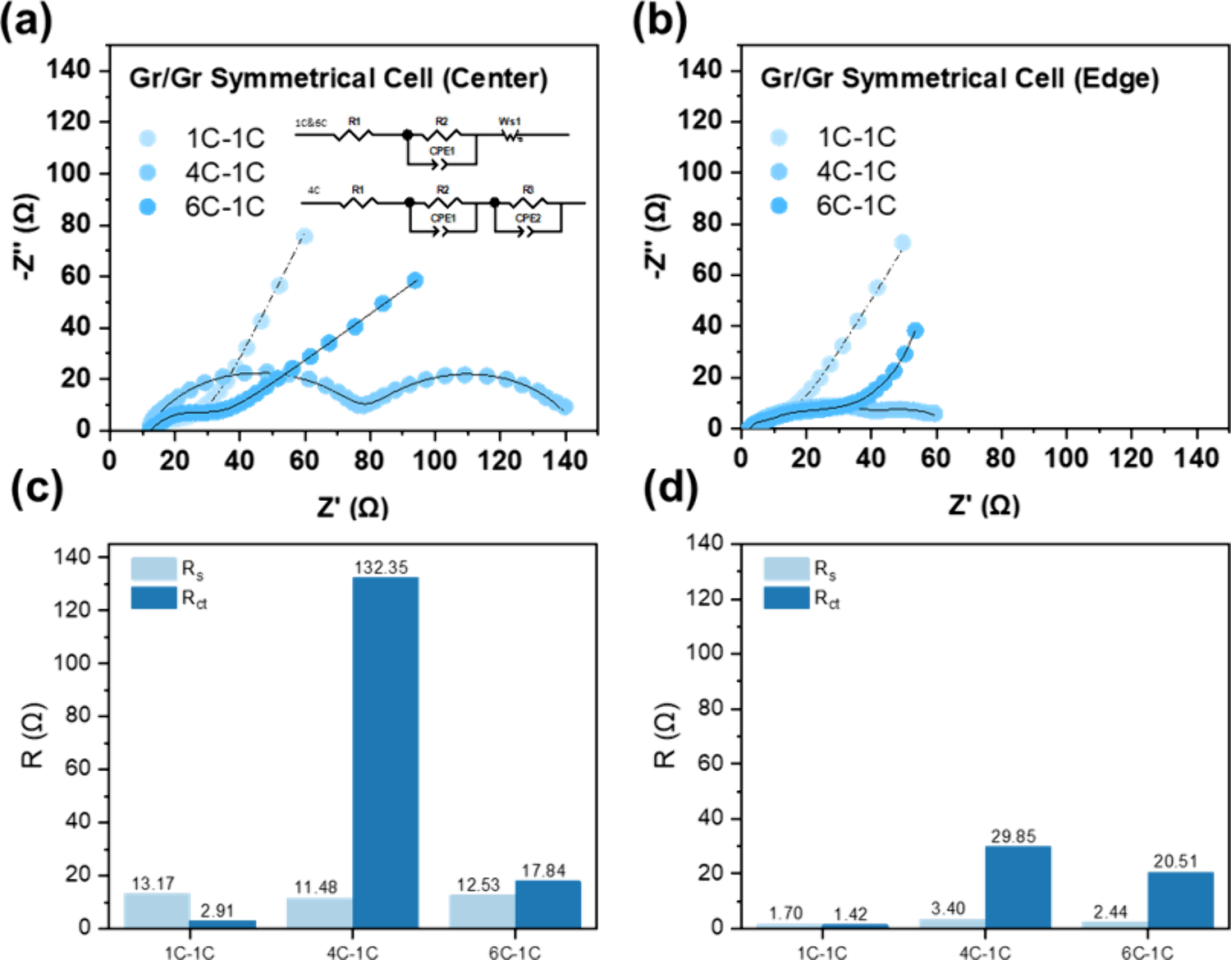
EIS Nyquist plots and interface impedance values of cycled Gr at (a, c) center and (b, d) edge after cycling at 1C-1C 1st cycle, 4C-1C, and 6C-1C.

These results are consistent with the Gr morphology and SEI thicknesses locally observed by both Cryo FIB-SEM and STEM-EDS, indicating that the cause of capacity decay at 4C is due to continuous SEI formation growth at the anode surface and bulk, resulting in inactive Li in the form of Li_*x*_Gr. Considering the thinner SEI and impedances associated with the 6C Gr anode, the cell was recycled after 80% capacity retention at a slower charge rate of 1C (Figure S14). While stable cycling is achieved, the delivered capacity is lower than a cell charged at 1C for an identical absolute number of cycles. This implies that while some capacity decay during fast charging is attributed to SEI formation on the anode, much of the capacity is reversible, pointing to the kinetically limited Li^+^ diffusion in Gr as a dominant mechanism for the capacity decay.

As fast charging generates ohmic heat because of extreme polarization, the underlying SEI may behave or age differently under elevated temperatures. It is commonly reported that continuous operation at elevated temperatures boosts interfacial reactivity, promoting growth of compact and less permeable SEI.^43–46^ Therefore, local surface temperature measurements were taken to corroborate the uneven temperature distribution to the difference in local degradation between center and edges of the Gr anode with charging rate. Optical sensors based on fiber Bragg gratings (FBG) were utilized due to their linear response to temperature, with their small size and flexibility being conducive to operando-monitoring of Li-ion batteries, as previously explored.^47,48^ Three FBG sensors are placed onto the 18650-cell casing with two placed symmetrically at the top and bottom of the cell, and one in between at the middle. The 18650 cell was subsequently subjected to galvanostatic cycling at increasing rates of charge. A 5-h relaxation period is introduced between every charge and discharge to allow for thermal equilibrium, as highlighted in Figure 5a. In conjunction with the voltage profiles, the peak cell temperature increases with increasing rates of charge, most prominently from 1C to 4C (*T*_center_ = 27.8 to 33.4 °C). Notably, the peak temperature associated with the charging process supersedes that of the discharge process beyond 1C, depicting the kinetic limitations of the Gr intercalation. As for 4C and 6C rates of charge, the peak cell temperature drops drastically, due to the rapid polarization and early termination of the charging step from hitting the cutoff voltage (3.6 V). Subsequent surface temperature measurements were conducted under identical cycling protocols to the cells characterized, where a constant voltage (CV) hold was introduced with no relaxation period. It is observed that the cell temperature peaks during the CV step for both charging rates 4C and 6C, as shown in Figure 5b,c. Locally, the temperature at the center of the cell is consistently higher with cycling at a charging rate of 4C (T_peak,center_ = 37.8 °C), approximately 1 °C higher than the edges of the battery (T_peak,top_ = 36.9 °C, T_peak,top_ = 36.7 °C). On the other hand, the temperature at the center of the cell is observed to be marginally, albeit consistently, lower than the edges at a charging rate of 6C (T_peak,bottom_ = 35.1 °C). As expected, temperature build up is seen from the heat generated during the charge process overlapping with the heat generated in the discharge process due to the lack of a rest interval. A near 5 °C difference is seen at a charging rate of 4C without rest, implicating the significant impacts of fast charging protocols. Previous reports^49^ have indicated that electrode geometry significantly influences lithiation performance. In particular, electrode edges were found to saturate rapidly and experience excessive lithium supply from the electrolyte, resulting in lithium dendrite formation under high lithium flux. Given that conventional jelly rolls feature graphite anodes longer than the cathodes, such edge effects may contribute to elevated local temperatures. Furthermore, Xiang Liu et al.^50^ reported SEI breakdown at temperatures as low as 40 °C, accompanied by lithium leaching from the Gr anode, and eventual reactions of lithium with the binder or SEI components above 100 °C. SEM characterization of the cycled separator along the electrode roll for the cell cycled at 4C shows an intact morphology (Figure S15), while global EDS results reveal phosphorus in the center of the jelly roll—originating solely from the electrolyte salt (LiPF_6_)—indicating electrolyte decomposition or SEI breakdown as opposed to separator-anode interactions at these relatively low temperatures (<50 °C). Although the measured surface temperatures remain below these thresholds, temperature buildup is expected to promote continuous SEI decomposition, and initiate Li^0^ plating over long-term cycling under these fast rates of charge. Based on the STEM-EDS, XPS and impedance characterizations in the above discussion, it is evident that both the temperature buildup and temperature difference between local areas of the cell are a direct contributor to the rate of SEI decomposition. Specifically, the pronounced temperature difference between the center and edges of the cell charged at 4C aligns with the extreme SEI buildup in the center of the Gr anode. While peak temperatures during the charging step were comparable, the relatively higher degree of temperature homogeneity and short-duration cycles directly resulted in a smaller difference between the SEI thickness locally. Given the observed correlation between SEI heterogeneity and thermal gradients, optimizing fast-charging protocols or implementing passive cooling through cell-level design is essential to balance battery health, safety, and longevity while minimizing charging time.

**Figure 5:**
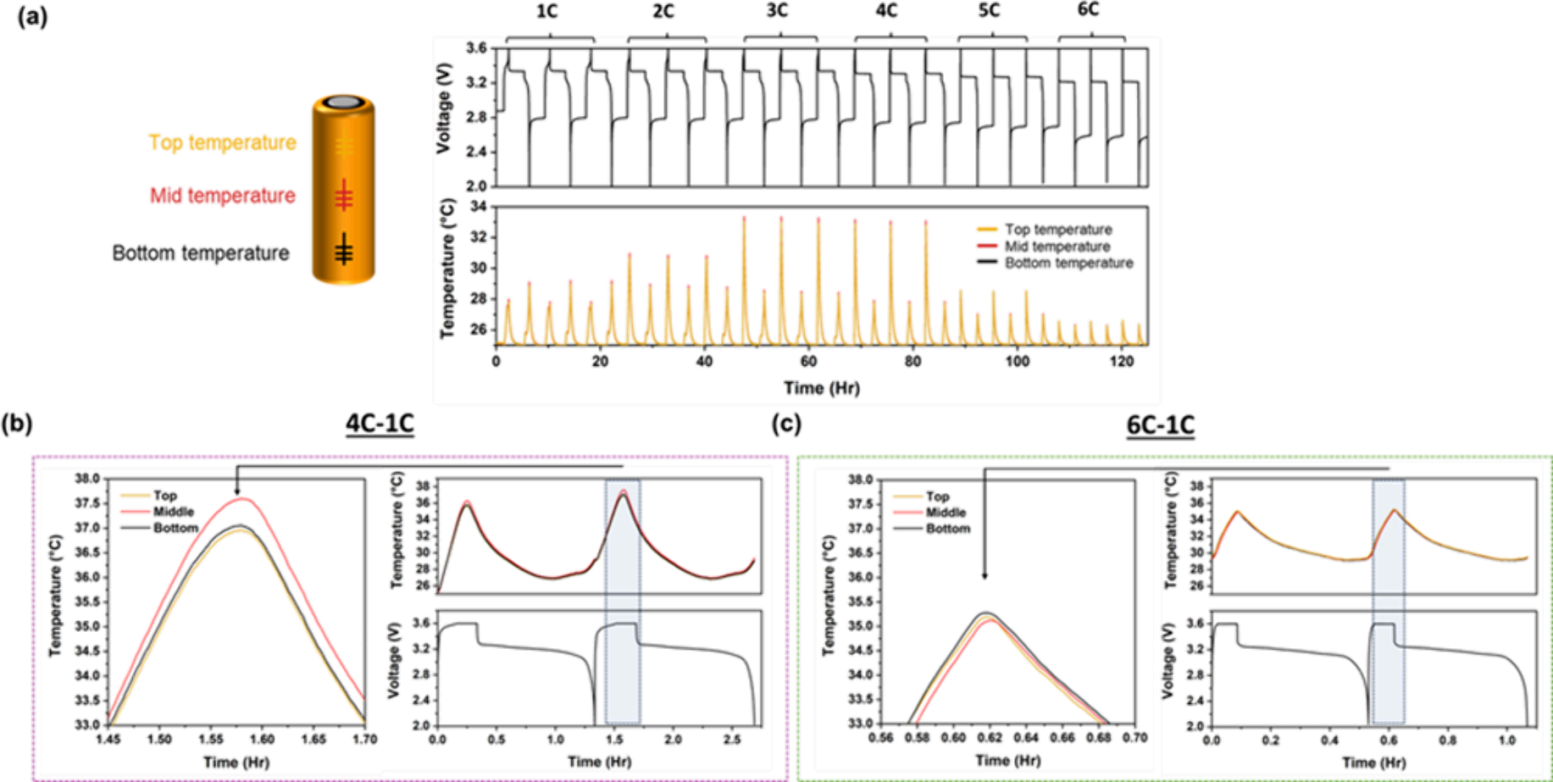
Localized surface temperature measurements via fiber Bragg grating optical sensors placed on top, middle and bottom of the cell case (a). Time resolved voltage and temperature profiles along with insets of highlighted regions depicted for (b) 4C-1C and (c) 6C-1C.

## Conclusion

In conclusion, the primary cause of capacity decay in commercial LFP/Gr cells under fast rates of charge was investigated and revealed to be arising from the excessive SEI formation, specifically considering rates of charge where Li^+^ diffusion across the interface and electrode are not initially the main rate-limiting steps (≤ 4C). XRD and FIB-SEM were applied to both the LFP cathodes and Gr anodes for bulk structural degradation, revealing more prominent degradation on the Gr anode. Spatial differences in the observed degradation on the Gr anode were observed between 4C and 6C rates of charge. Subsequently, TGC was applied to the Gr anodes to differentiate between plated Li^0^ and inactive Li_*x*_C_6_. Although both sources of Li were comparable between the center and edge of the electrodes at 4C or 6C, EIS results revealed significant differences in impedances spatially and between the two rates of charge. Cycle-life-induced degradation between center and edges of the Gr anode differed for 4C and 6C rates of charges, owing to the kinetically limited Li^+^ diffusion at 6C whereas the primary degradation mechanism at 4C is continuous SEI formation. TEM and XPS characterization of the anodes and cathodes post-mortem support these results. In addition, local surface temperature measurements align with the discrepancy in rate of SEI formation not only spatially, but between 4C and 6C rates of charge. Peak surface temperature differences between the Gr anode center and edge were found to be 1.0 and 0.1 °C at 4C and 6C, respectively. Likewise, the SEI thickness between the Gr anode center and edge was found to be ≥700 μm for 4C and ∼100 μm for 6C cycling. Clearly, the increased temperature over long cycling incurs a higher rate of SEI formation, highlighting the importance of effective thermal management. We believe that this study provides a unique understanding of the primary cause of capacity decay under fast charging conditions in 18650 LFP/Gr cells, highlighting both comprehensive understanding of the lithium inventory and appropriate thermal management as crucial strategies in achieving longer lifetimes of secondary batteries targeting fast-charging applications.

## Supplementary Material



## Notes

Additional optical and SEM images, XRD, XPS, TEM, STEM-EDS, ICP-MS and EIS results of the electrodes characterized. ^19^F, ^31^P, ^13^C, and ^1^H NMR spectra for the system electrolyte. Additional cycling data and tabulated electrode thicknesses. Experimental methods for titration gas chromatography and calibration curve. (PDF)
