# Supplementary material for: Unveiling the Graphite Electrolyte Interphase Evolution under Fast Charging Conditions in Commercial Cells
Source: ACS Appl Mater Interfaces. 2025 Dec 2;17(50):68650–60. doi: 10.1021/acsami.5c17267 (PMC12723637; doi:10.1021/acsami.5c17267)
Supplement: Supplementary file 1 [file am5c17267_si_001.pdf]

Supporting Information

Unveiling the Graphite Electrolyte Interphase Evolution Under Fast Charging Conditions in Commercial Cells

Alex Liu[1], Weikang Li[1], Bing Han[1], Phillip Ridley[1], Louis Ah[1], Bhargav Bhamwala[1], Marta Vicencio[1], Dhevathi R.R. Kannan[3], Vallabha R. Rikka[3], Vinay Premnath[3], Judith A. Jeevarajan[3], Wurigumula Bao[2]*, Ying Shirley-Meng[1,2]*s

[1]Aiiso Yufeng Li Family Department of Chemical and Nano Engineering, University of California, San Diego, La Jolla, CA 92093, USA

[2]Pritzker School of Molecular Engineering, University of Chicago, Chicago, IL 60637, USA

[3]Underwriters Laboratories Research Institutes, Electrochemical Safety Research Institute, Houston, TX 77204, USA

Correspondence to:

- wubao@uchicago.edu
- shirleymeng@uchicago.edu

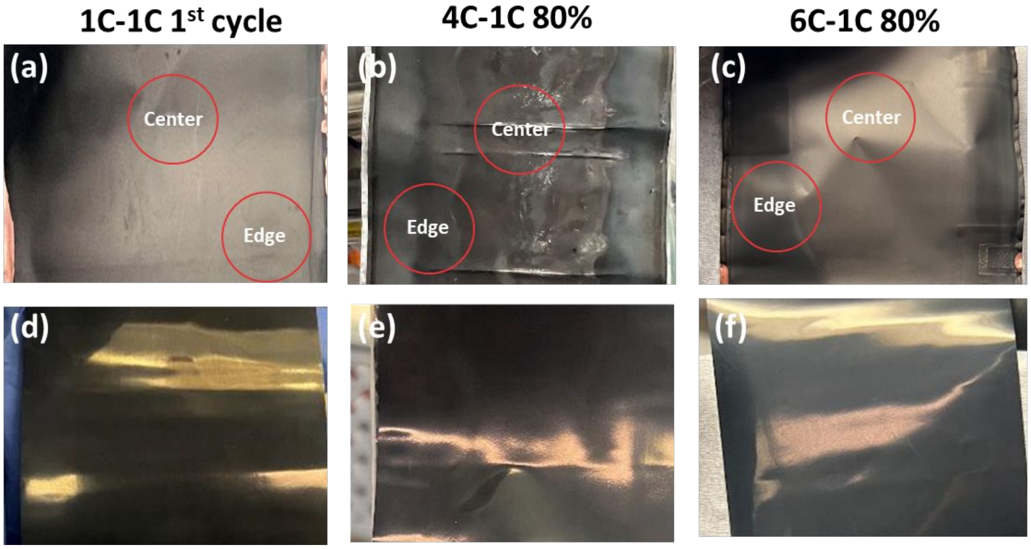

Figure S1. Optical images of (**a**-**c**) Gr anode and (**d**-**f**) LFP cathode cycled at 1C-1C 1st cycle, 4C-1C, and 6C-1C.

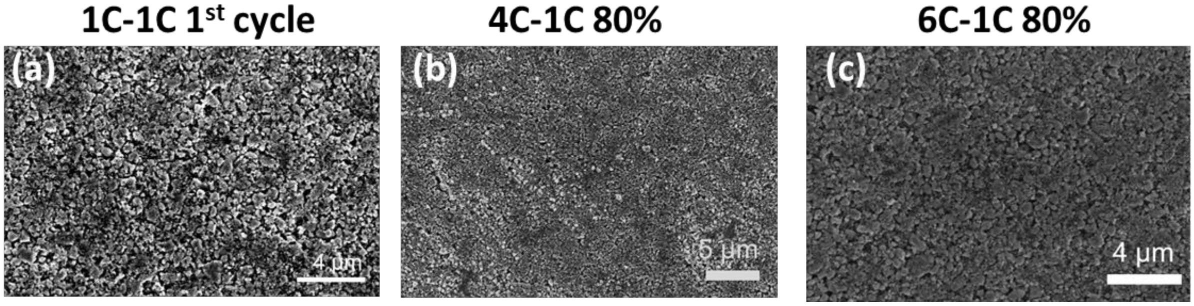

Figure S2. Surface SEM images of LFP cathode cycled at (**a**) 1C-1C 1st cycle, (**b**) 4C-1C, and (**c**) 6C-1C

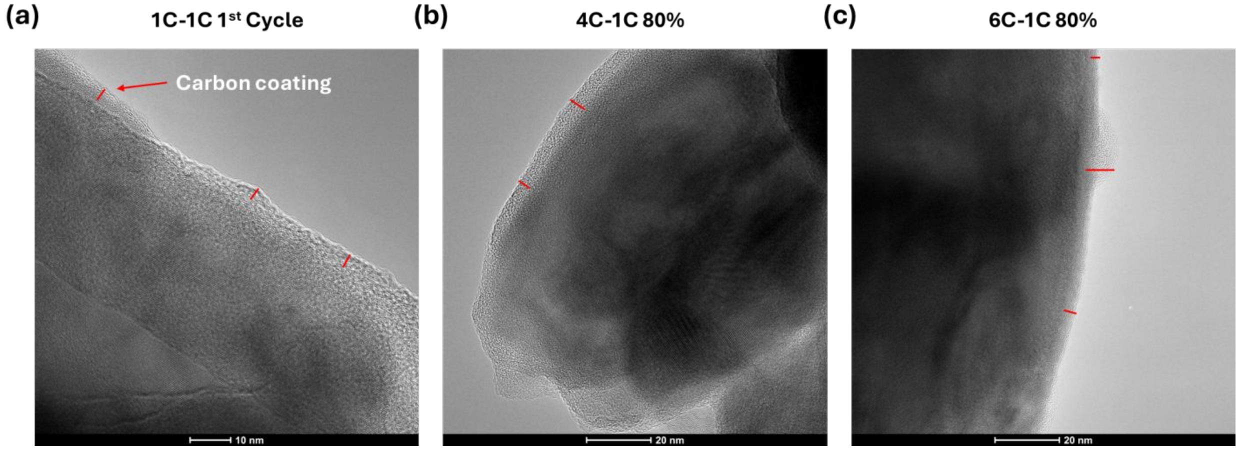

Figure S3. TEM images of LFP particles after (**a**) 1C-1C 1st cycle, (**b**) 4C-1C, and (**c**) 6C-1C. Red lines depict thickness of the amorphous carbon coating.

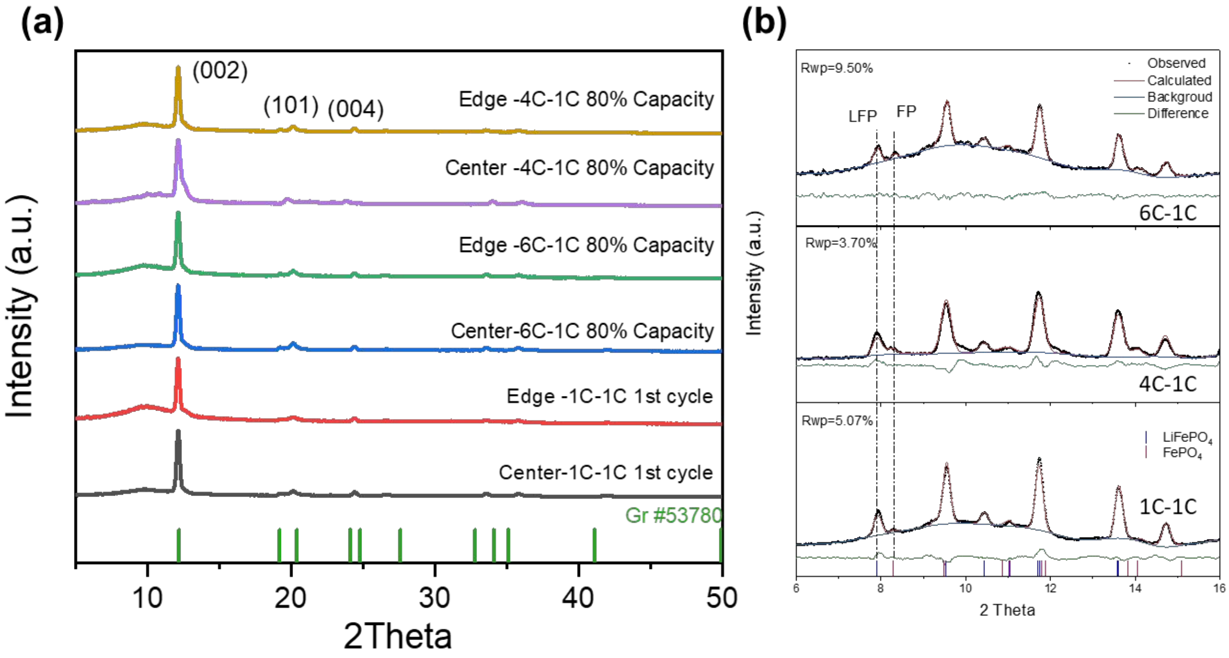

Figure S4. (**a**) Center and edge XRD spectra of cycled Gr at 1C-1C 1st cycle, 4C-1C, and 6C-1C. (**b**) XRD spectra of cycled LFP at 1C-1C 1st cycle, 4C-1C, and 6C-1C.

Table S1. Reactions of metallic lithium and lithiated graphite with protic solvent (H_2O)

Anode	Reaction	Li:H_2
Li	$Li + H_2O \rightarrow LiOH + x/2H_2$	2:1
C	$Li_xC_6 + xH_2O \rightarrow xLiOH + C_6 + x/2H_2$	2:1

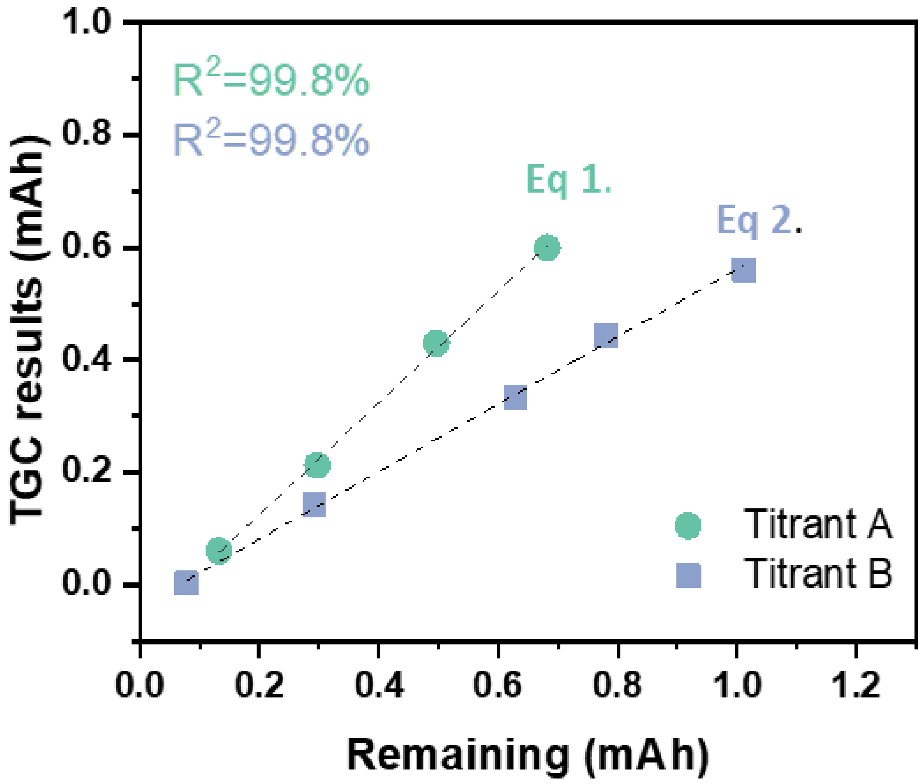

Figure S5. Titration gas chromatography calibration curve for tracking of Li-inventory within cycled graphite anodes in H_2O + 3 M H_2SO_4 (Titrant A) and H_2O (Titrant B)

A linear relationship is established and verified utilizing H_2O titrant only in addition to H_2O followed by 3 M H_2SO_4 as depicted with Eq 2. and Eq. 1, respectively. Eq. 2 describes the H_2 evolution based on fully titrated Li^0 and partially titrated Li_xC_6, and Eq. 1 describes the H_2 evolution based on fully titrated Li^0 and Li_xC_6. The difference between Eq. 1 and Eq. 2 yields the relationship between partial and total Li_xC_6:

$$Li_xGr(Partial - mAh) = 0.597 \times Li_xGr(Total - mAh) - 0.044$$

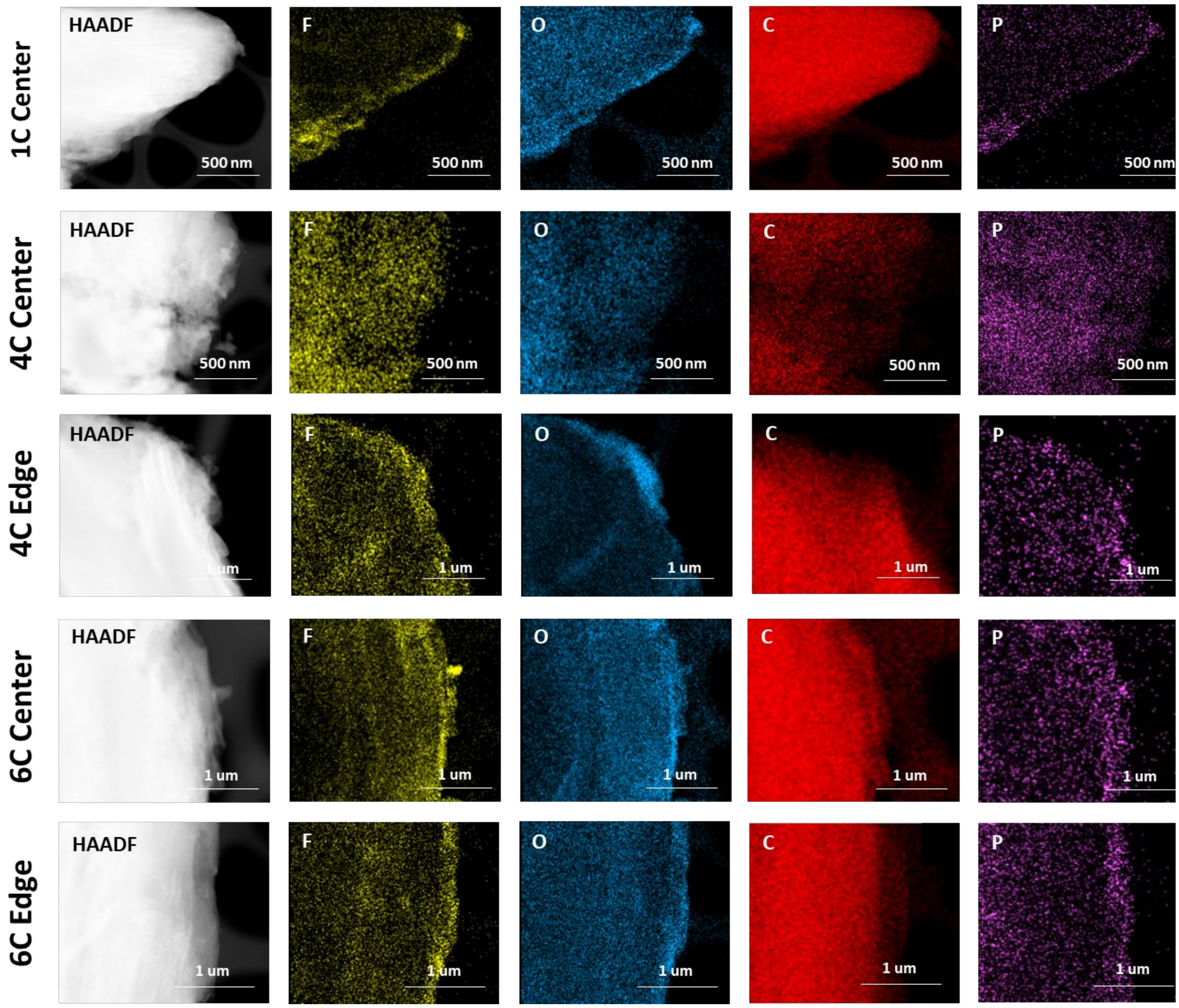

Figure S6. Summarized STEM-EDS results (C, O, F, P) of the cycled Gr anodes for center and edge at various rates of charging: 1C-1C 1st cycle; 4C-1C 80%; 6C-1C 80%

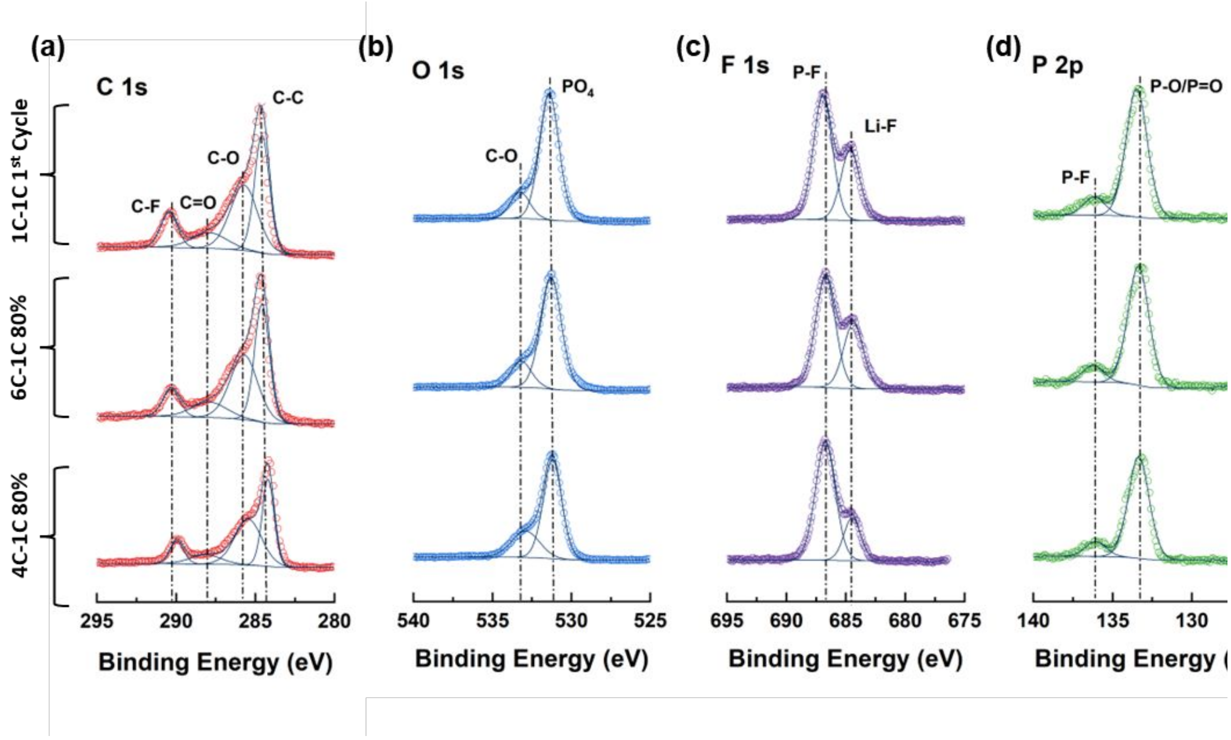

Figure S7. XPS spectra of the cycled LFP at 1C-1C 1st cycle, 4C-1C, and 6C-1C where the spectra (**a-d**) correspond to C 1s, O 1s, F 1s, and P 2p, respectively.

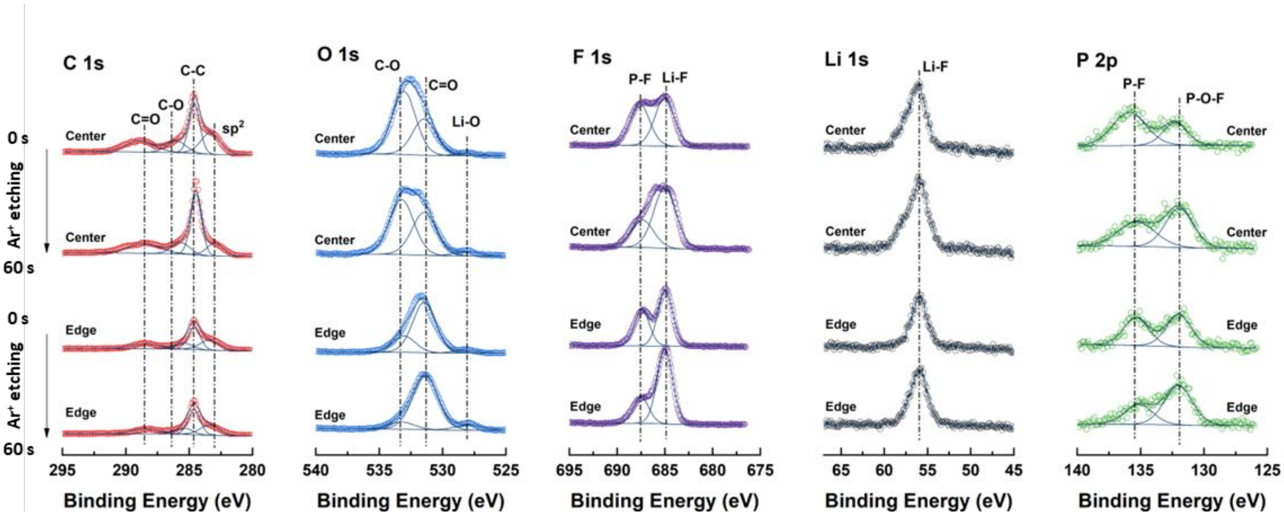

Figure S8. XPS spectra (C 1s, O 1s, F 1s, Li 1s, P 2p) of cycled Gr anode (1C-1C 1st cycle)

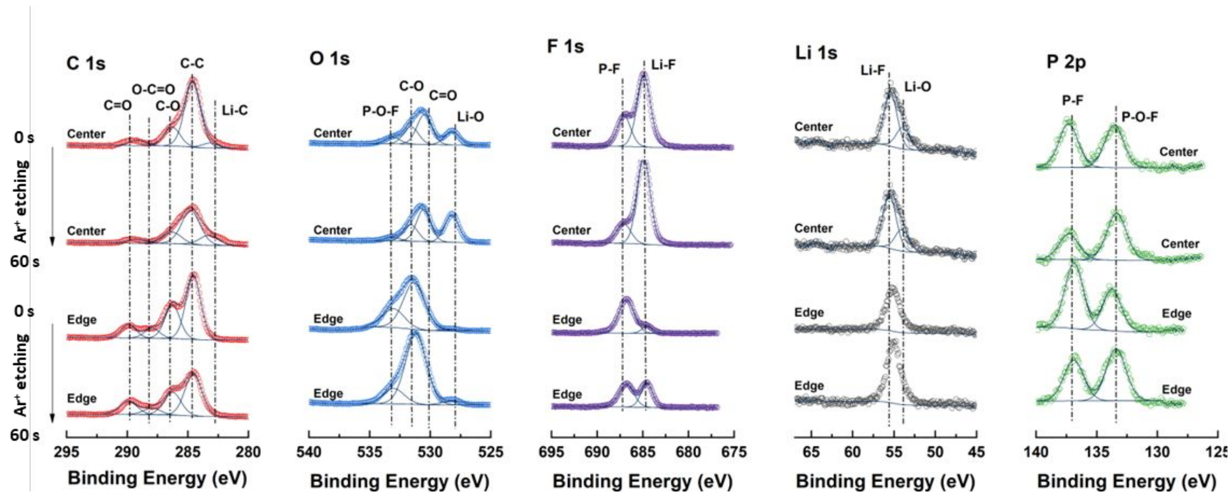

Figure S9. XPS spectra (C 1s, O 1s, F 1s, Li 1s, P 2p) of cycled Gr anode (4C-1C 80%)

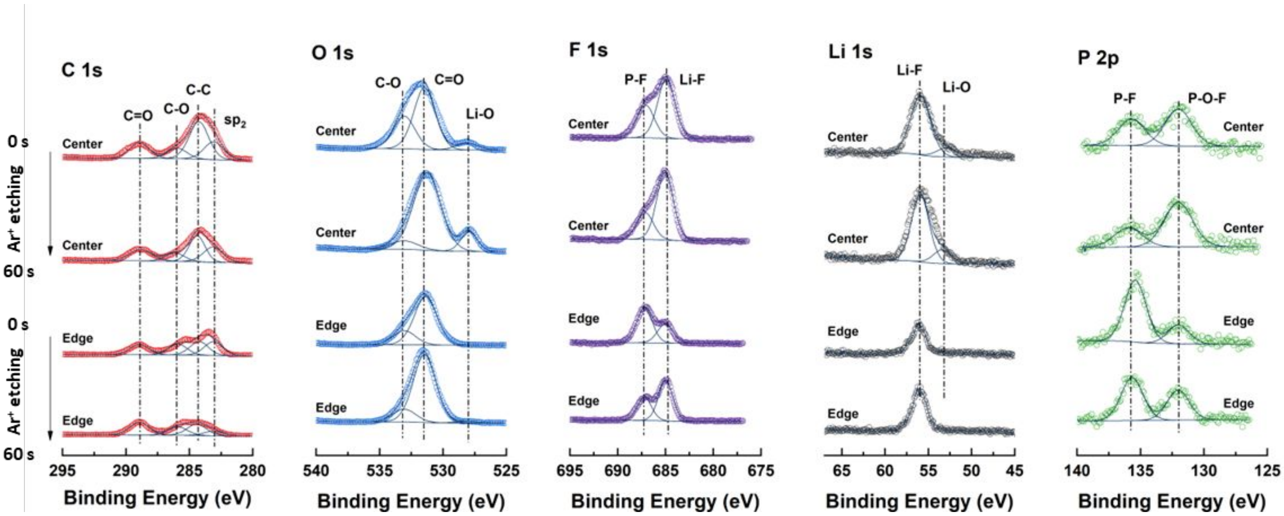

Figure S10. XPS spectra (C 1s, O 1s, F 1s, Li 1s, P 2p) of cycled Gr anode (6C-1C 80%)

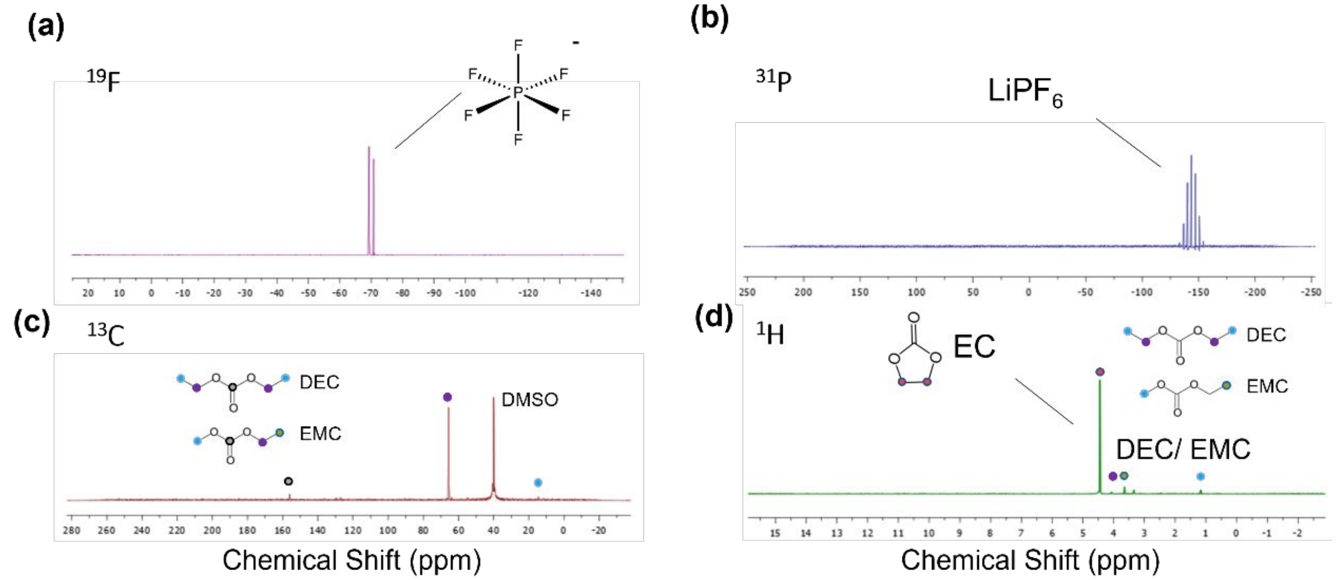

Figure S11. (**a**) ^{19}F, (**b**) ^{31}P, (**c**) ^{13}C and (**d**) ^{1}H NMR spectra of electrolyte from the commercial Gr-LFP cell

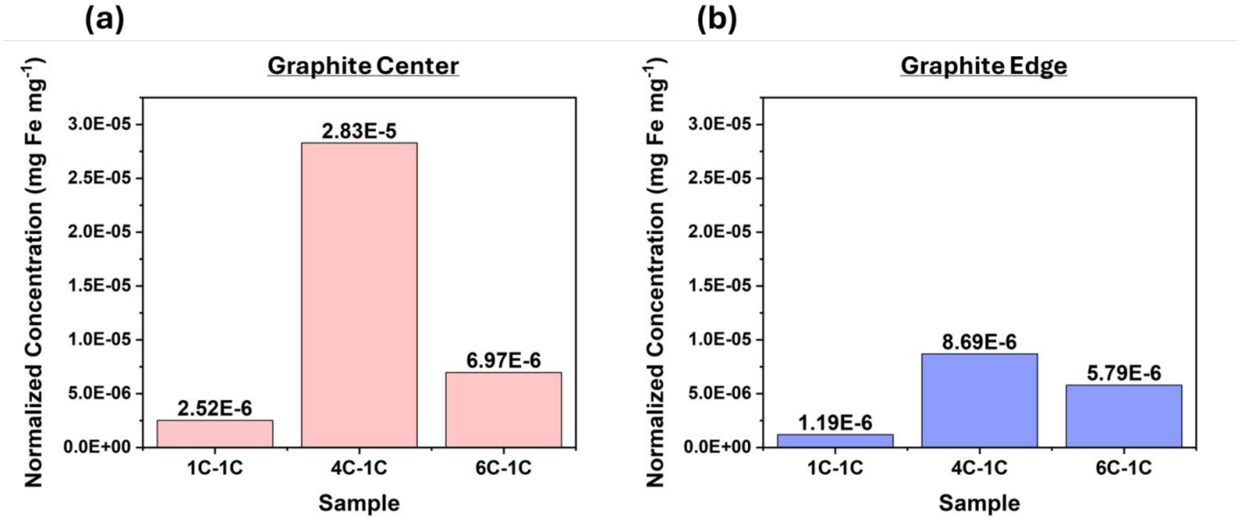

Figure S12. Normalized Fe concentration by ICP-MS on cycled Gr anode across 1C-1C 1st cycle, 4C-1C 80%, and 6C-1C 80% in the electrode (**a**) center and (**b**) edge

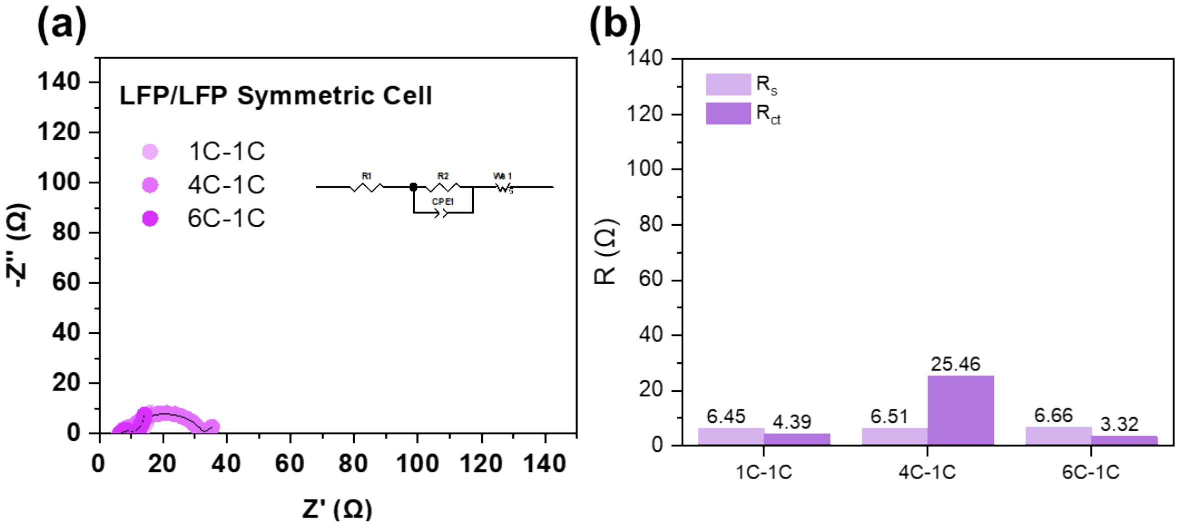

Figure S13. (**a**) EIS Nyquist plots and (**b**) fitted impedance values of cycled LFP at 1C-1C 1st cycle, 4C-1C, and 6C-1C.

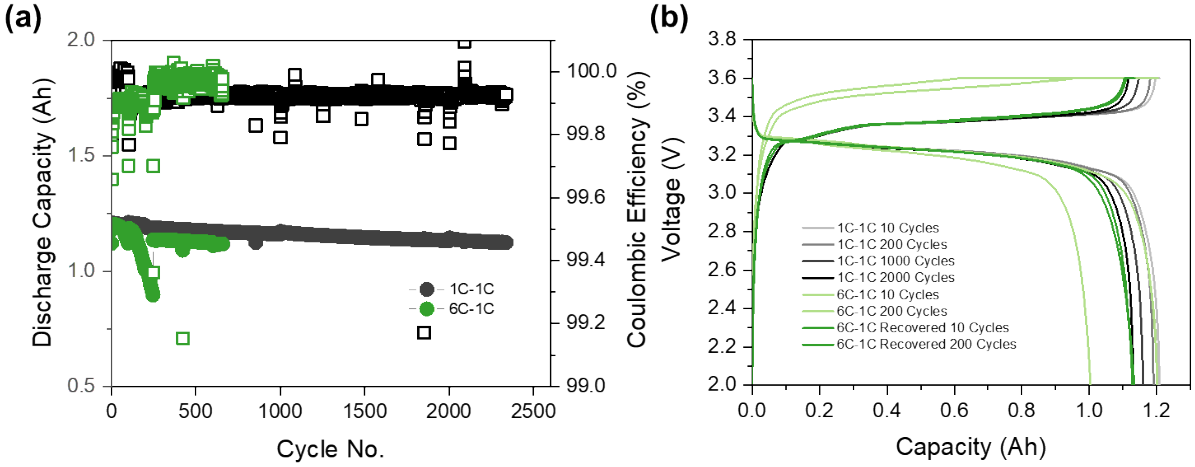

Figure S14. (**a**) Cycling performance and (**b**) charge-discharge curve of 6C-1C cell after cycling at 1C-1C.

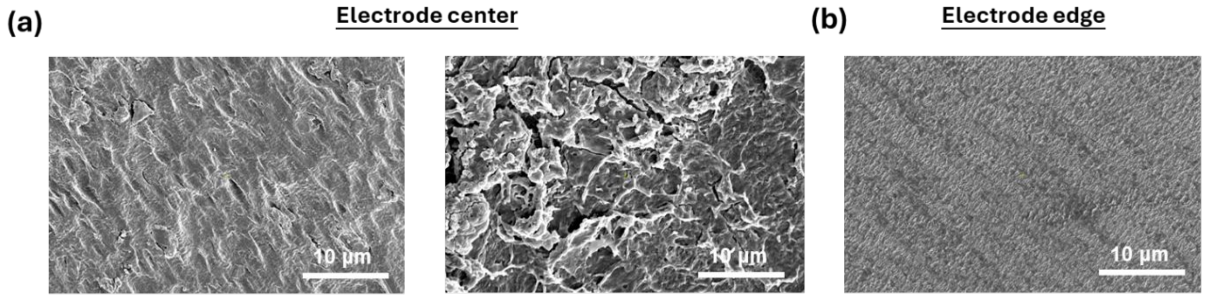

(c)

Element	Atomic %
C	80.75
O	13.58
F	5.05
P	0.62

Element	Atomic %
C	72.96
O	18.23
F	7.17
P	1.63

(d)

Element	Atomic %
C	84.41
O	15.59
F	0.00

Figure S15. Surface SEM images of the cycled separator at 4C-1C 80% in the (**a**) center and (**b**) edge. Corresponding EDS results of the separator in the (**c**) center and (**d**) edge.

Table S2. Thickness of Gr anode at center and edge at various rates of charge

Thickness of Gr electrode	Center (um)	Edge (um)
1C-1C 1st cycle	85	85
6C-1C at 80% capacity retention	85	90
4C-1C at 80% capacity retention	129	91
